# Time to positivity of *Corynebacterium* in blood culture: Characteristics and diagnostic performance

**DOI:** 10.1371/journal.pone.0278595

**Published:** 2022-12-13

**Authors:** Naoki Watanabe, Yoshihito Otsuka, Tomohisa Watari, Naoto Hosokawa, Kazufumi Yamagata, Miyuki Fujioka

**Affiliations:** 1 Department of Clinical Laboratory, Kameda Medical Center, Kamogawa, Chiba, Japan; 2 Graduate School of Health Sciences, Hirosaki University, Hirosaki, Aomori, Japan; 3 Department of Infectious Diseases, Kameda Medical Center, Kamogawa, Chiba, Japan; Stellenbosch University, SOUTH AFRICA

## Abstract

The presence of *Corynebacterium* in blood samples can indicate true bacteremia or contamination, thus complicating the diagnosis of true bacteremia. We aimed to evaluate the usefulness of time to positivity (TTP) in diagnosing true bacteremia and contamination in cases where *Corynebacterium* was isolated from blood samples. We compared the TTP of the true-bacteremia group (n = 77) with that of the contamination group (n = 88). For the true-bacteremia cases that had only one set of positive blood cultures (n = 14), considering clinical and bacteriological data, additional cultures were performed on blood or other specimens. The same *Corynebacterium* spp. as in blood were isolated from these specimens. Receiver operating characteristic curves were generated, and the sensitivity and specificity of TTP were calculated for diagnosing true bacteremia. The median TTP of the true-bacteremia group (26.8 h) was shorter than that of the contamination group (43.3 h) (P < 0.0001). When considering TTP ≤ 25.0 h as true bacteremia, the sensitivity and specificity were 44.2% and 95.5%, respectively. Moreover, when considering TTP ≤ 69.4 h as true bacteremia, the sensitivity and specificity were 96.1% and 20.5%, respectively. Among the true-bacteremia groups with one set of positive blood cultures (n = 14), no case exhibited a TTP > 69.4 h. Only three cases showed TTP ≤ 25.0 h in the true-bacteremia group with one set of positive blood cultures. TTP > 69.4 h is likely to indicate contamination and may be useful to exclude true bacteremia in cases with one set of positive blood cultures. Meanwhile, diagnosing true bacteremia using the threshold of TTP 25.0 h would be difficult. Therefore, the clinical and bacteriological data are important for diagnosing bacteremia, especially in cases with TTP ≤ 69.4 h.

## Introduction

Coryneform bacteria are irregular gram-positive rods which encompass a number of genera of which *Corynebacterium* is the most clinically encountered [1]. *Corynebacterium* causes opportunistic infections in immunocompromised patients, including those with artificial devices, or in long-term hospitalizations, which is an issue of increasing concern [2, 3]. The presence of *Corynebacterium* typically indicates contamination when isolated from a clinical specimen, such as blood and wound samples. However, when this occurs, it can be challenging to determine whether the strain is a contaminant or a true pathogen [4]. Additionally, true bacteremia or contamination also depends on the isolated species. For example, Yamamuro *et al*. [5] reported higher rates of true bacteremia with *C*. *striatum* (70%) and *C*. *jeikeium* (70%) than other *Corynebacterium* spp. (9%). However, the presence of *C*. *striatum* and *C*. *jeikeium* in blood samples can be due to contamination, which also applies to other *Corynebacterium* species.

Previous studies have demonstrated that time to positivity (TTP), which is the time from the start of blood culture to a positive result, may be helpful in distinguishing between true bacteremia and contamination [6, 7]. However, no studies have reported sufficient data on *Corynebacterium*. Therefore, we evaluated the usefulness of TTP to distinguish true bacteremia from contamination in cases where *Corynebacterium* was isolated from blood samples. Lipophilic *Corynebacterium* spp. require an extended period to recover in broth media [1]. Furthermore, Ruiz-Giardín *et al*. [7] reported that the TTP of patients receiving antibiotics within one week prior to blood-culture collection (14 h) was longer than that of patients who did not receive any antibiotics (12 h). Hence, we also assessed the effects of the *Corynebacterium* spp. group and history of antibiotic administration on TTP. This is the first study to characterize the TTP of *Corynebacterium*. If TTP is found to be useful in distinguishing true bacteremia from contamination in cases positive for *Corynebacterium*, it will help clinicians to achieve a diagnosis of true bacteremia. Therefore, an accurate diagnosis of either true bacteremia or contamination will help patients to receive appropriate medical care and enable proper use of antibiotics.

## Methods

### Study design

In this retrospective study, we compared the TTP of the true-bacteremia group and contamination group in cases where *Corynebacterium* was isolated from blood cultures. The following aspects were evaluated: (1) comparison of TTP between the true-bacteremia and contamination groups, (2) comparison of TTP between antibiotic use and no antibiotic groups, (3) comparison of TTP between the non-lipophilic and lipophilic *Corynebacterium* spp. groups, and (4) whether TTP is an independent factor affecting the diagnosis group (true-bacteremia or contamination groups).

### Patient eligibility and selection

This study was conducted at the Kameda Medical Center, Japan, which is an 865-bed tertiary care hospital that provides general medicine, surgery, and hematopoietic stem cell transplantation services. This study was approved by the Research Ethics Committee of the Kameda Medical Center (approval no. 21-138-220510) and was performed in agreement with the Declaration of Helsinki. Furthermore, this study included only anonymized data. The need for written informed consent from the participants of this study was waived by the Research Ethics Committee of the Kameda Medical Center.

This study included cases with positive blood cultures and *Corynebacterium* isolated between January 2014 and February 2022. Of the 216 cases in which *Corynebacterium* was isolated. Multiple bacterial species other than *Corynebacterium* had the potential for growth that affected TTP. To avoid this effect, 51 patients with bacteremia caused by multiple bacterial species were excluded. We collected the following data of 165 patients from electronic medical records: age, sex, inpatient or outpatient status, department, history of antibiotic administration (antibiotic use or no antibiotic group), and results of microbiological tests (number of positive blood-culture sets, TTP, species of *Corynebacterium*, and culture results of each specimen). TTP was defined as the time from the loading of blood-culture vial into the Bactec FX system (BD, Franklin Lakes, NJ, USA) up to the time when the detection signal became positive. TTP was first collected on blood cultures with *Corynebacterium*.

Only the first positive blood-culture set was included in the study when a patient had multiple positive results. When the same patient had multiple positive blood-culture results and blood cultures were collected at different times over two weeks, they were considered different episodes.

### Microbiological methods

Blood cultures were performed on patients with suspected sepsis based on the medical criteria. Two sets of blood cultures (aerobic and anaerobic culture vials each) were collected from each patient. Subsequently, blood cultures were performed using culture vials (Plus Aerobic medium, Lytic Anaerobic medium, Peds Plus medium for pediatric, and Myco/F Lytic medium, BD, Franklin Lakes, NJ, USA) and Bactec FX system. Incubation condition was 35°C for 7 days, except for Myco/F Lytic medium, which was 35°C for 35 days.

From January 2014 to May 2015, *Corynebacterium* identification was performed using RapID CB Plus (Remel, Lenexa, KS). From June 2015 to February 2022, the identification was performed using matrix-assisted laser desorption ionization-time of flight mass spectrometry (MALDI-TOF MS; Bruker Biotyper, Bruker Daltonics, Inc., Billerica, MA). *Corynebacterium* groups were classified as non-lipophilic or lipophilic based on the Manual of Clinical Microbiology, 12th Edition [8]. For the classification of the *Corynebacterium*, *C*. *afermentans* (*C*. *afermentans* subsp. *afermentans* or *C*. *afermentans* subsp. *lipophilum*) and other bacteria were excluded from the analysis.

### Definitions

We identified true bacteremia in cases that fulfilled the following inclusion criteria: (1) two sets of blood cultures were positive and the same *Corynebacterium* species was isolated, or (2) the same *Corynebacterium* species was isolated from one set of blood cultures and at least one other specimen from the site suspected of infection, in addition to the clinical symptoms. Contamination was determined in cases that met the following criteria: (1) only one set of blood cultures was positive and the culture results with other clinical samples were negative for *Corynebacterium*, or (2) only one set of blood cultures was positive, and the infection was likely caused by a pathogen other than *Corynebacterium*.

In the cases of clinically suspected true bacteremia with only one set of positive blood cultures, additional blood cultures were performed. If those blood cultures were positive and the *Corynebacterium* species detected was the same as that in the primary blood cultures, such cases were considered true bacteremia [5, 9].

### Statistical analysis

Statistical analysis was performed using EZR (software that extends the capabilities of R and R Commander) [10]. Continuous variables are presented as mean ± SD or median and interquartile range (IQR). Furthermore, they were tested for normality using the Kolmogorov–Smirnov test and were considered not normally distributed when *P* < 0.05. Categorical variables were presented as numbers and percentages (%).

The relationship between the diagnosis group and age was evaluated using Student’s *t* test (independent samples, two-sided). The relationship between the diagnosis group and categorical variables was evaluated using Fisher’s exact test. Categorical variables included the diagnosis group, inpatient or outpatient status, hematology department, emergency department, intensive care unit, and history of antibiotic administration. The relationship between TTP and categorical variables was evaluated using the Mann–Whitney U test (two-sided). Categorical variables included diagnosis group, history of antibiotic administration, and *Corynebacterium* group. Species-level identification of *Corynebacterium* can be difficult when using RapID CB Plus system. Therefore, we compared TTP between the RapID CB Plus and MALDI-TOF MS duration to evaluate the impact of different identification methods on TTP.

We created a receiver operating characteristic (ROC) curve and determined the sensitivity, specificity, and accuracy for the diagnosis of true bacteremia in cases with TTP below a set threshold. The accuracy was calculated as follows: [(sensitivity) × (prevalence) + (specificity) × (1—prevalence)]. Prevalence was defined as the percentage of cases in the true-bacteremia group among all the analyzed cases. We evaluated the association between the main variables (diagnosis group, *Corynebacterium* group, and history of antibiotic administration) and below-threshold TTP (TTP at which the maximum value was obtained for the sum of sensitivity and specificity). Additionally, we evaluated the effectiveness of the TTP threshold in diagnosing true-bacteremia cases with only one set of positive blood cultures.

Multivariate logistic regression analysis was performed as follows. The case with below-threshold TTP obtained from the ROC curve was used as the objective variable (the value at which sensitivity and specificity were maximized); age, sex, department, diagnosis group, history of antibiotic administration, and *Corynebacterium* group were considered as the explanatory variables. Variables with a *P* value of 0.5 or higher were excluded and recalculated.

All statistical methods were considered statistically significant when *P* < 0.05.

## Results

### Clinical characteristics and *Corynebacterium* spp. in the blood-culture positive cases

Of the 165 cases evaluated, 77 (46.7%) belong to the true-bacteremia group, and 88 (53.3%) were from the contamination group (Table 1). Of the 165 blood-culture positive cases in which *Corynebacterium* was isolated, non-lipophilic *Corynebacterium* was isolated in 122 cases (73.9%), and lipophilic *Corynebacterium* in 26 cases (15.8%; Table 2). Lipophilic properties of the strains isolated from the remaining 17 cases (10.3%) could not be defined (lipophilic-unknown *Corynebacterium*). The most frequently isolated species was *C*. *striatum* (52.1%, 86/165) followed by *C*. *jeikeium* (10.3%, 17/165).

**Table 1:** Clinical characteristics of cases with *Corynebacterium-*positive blood cultures.

Characteristics	Total No. (%)	True-bacteremia group No. (%)	Contamination group No. (%)	*P*
	*n* = 165	*n* = 77	*n* = 88	
Age in years, median ± SD	70.1 ± 15.0	70.0 ± 13.1	70.3 ± 16.5	0.89
Female	50 (30.3)	23 (29.9)	27 (30.7)	1
Inpatient	114 (69.1)	63 (81.8)	51 (58.0)	0.001
Hematology department	59 (35.8)	40 (51.9)	19 (21.6)	< 0.0001
Emergency department	38 (23.0)	8 (10.4)	30 (34.1)	< 0.001
Intensive care unit	13 (7.9)	3 (3.9)	10 (11.4)	0.09
Two sets of culture positive	63 (38.2)	63 (81.8)	0 (0)	Not determined
Antibiotic use group	88 (53.3)	55 (71.4)	33 (37.5)	< 0.0001

SD, standard deviation. *P* < 0.05 was considered statistically significant.

**Table 2:** *Corynebacterium* isolated from blood.

Group	Total no. (%)	True-bacteremia group no. (%)	Contamination group no. (%)
	*n* = 165	*n* = 77	*n* = 88
Non-lipophilic group	122 (73.9)	62 (80.5)	60 (68.2)
*C*. *striatum*	86 (52.1)	57 (74.0)	29 (33.0)
*C*. *amycolatum*	7 (4.2)	1 (1.3)	6 (6.8)
*C*. *minutissimum*	4 (2.4)	0 (0)	4 (4.5)
*C*. *aurimucosum*	2 (1.2)	0 (0)	2 (2.3)
*C*. *simulans*	2 (1.2)	0 (0)	2 (2.3)
*C*. *singular*	2 (1.2)	0 (0)	2 (2.3)
*C*. *riegelii*	1 (0.6)	1 (1.3)	0 (0)
*C*. *coyleae*	1 (0.6)	0 (0)	1 (1.1)
*C*. *mucifaciens*	1 (0.6)	0 (0)	1 (1.1)
Other species ^a^	16 (9.7)	3 (3.9)	13 (14.8)
Lipophilic group	26 (15.8)	14 (18.2)	12 (13.6)
*C*. *jeikeium*	17 (10.3)	12 (15.6)	5 (5.7)
*C*. *resistens*	5 (3.0)	1 (1.3)	4 (4.5)
*C*. *tuberculostearicum*	3 (1.8)	0 (0)	3 (3.4)
*C*. *urealyticum*	1 (0.6)	1 (1.3)	0 (0)
Lipophilic-unknown group	17 (10.3)	1 (1.3)	16 (18.2)
*C*. *afermentans* ^b^	6 (3.6)	0 (0)	6 (6.8)
*Corynebacterium* spp. ^c^	11 (6.7)	1 (1.3)	10 (11.4)

^a^ The following species were included; *Arcanobacterium haemolyticum* (true bacteremia: 1 case), *Brevibacterium* spp. (true bacteremia: 1 case, contamination: 4 case), *Dermabacter hominis* (contamination: 7 case), *Rothia dentocariosa* (contamination: 1 case), and *Trueperella bernardiae* (true bacteremia: 1 case, contamination: 1 case).

^b^ Strains for which *C*. *afermentans* subsp. *afermentans* or *C*. *afermentans* subsp. *lipophilum* could not be identified.

^c^ The strain was reported to be *Corynebacterium* because the species could not be identified.

### Comparison of TTP between true-bacteremia and contamination groups

**Fig 1:**
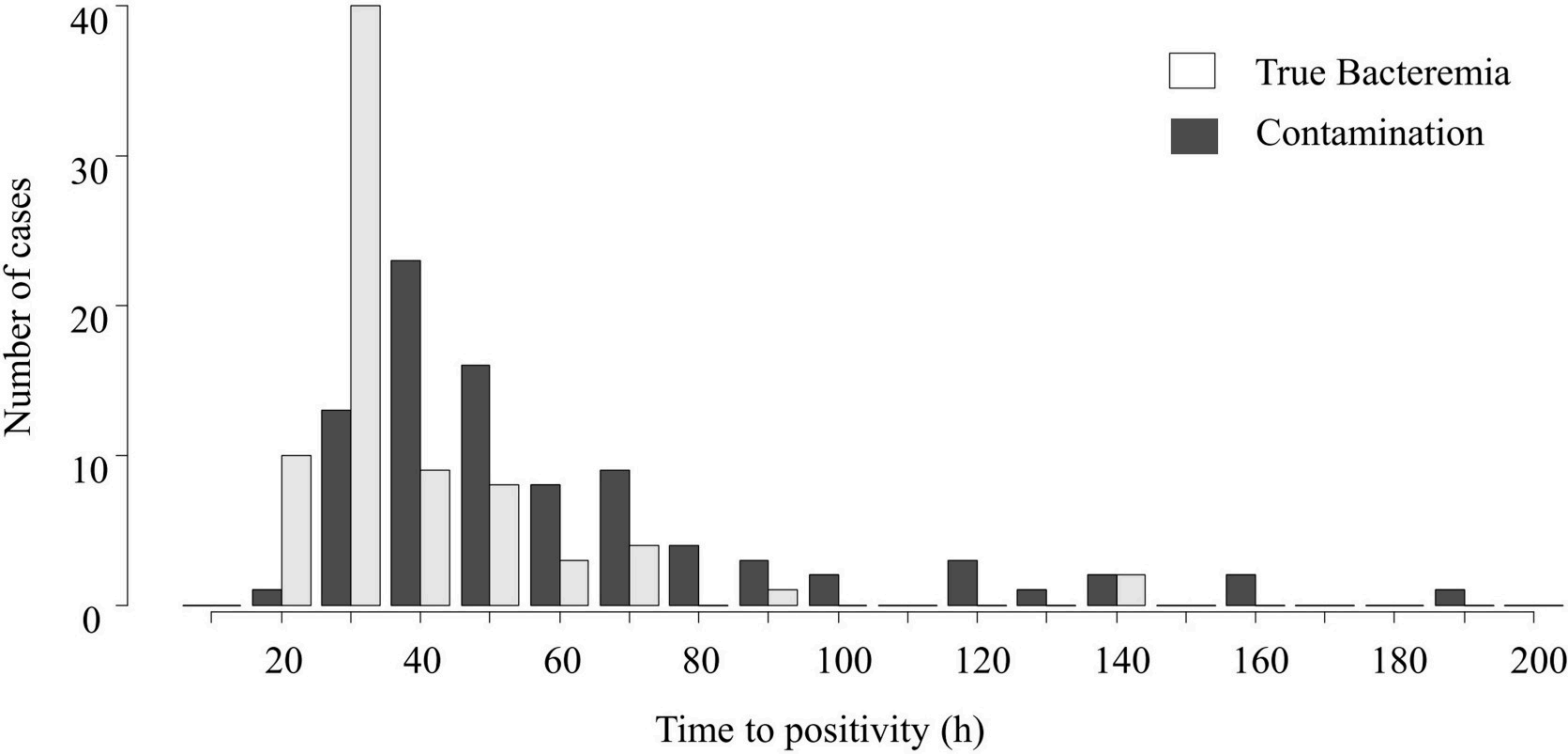
Number of cases per time to positivity. The figure shows the number of cases in time to positivity every 10 h. The numbers on the x-axis refer to the 10-h intervals.

Fig 1 shows the number of cases per TTP value. TTP did not follow a normal distribution (*P <* 0.05). The median TTP of the true-bacteremia group was 26.8 h (*n* = 77, interquartile range (IQR) = 23.0–38.2 h) (Table 3). The median TTP for the contamination group was 43.3 h (*n* = 88, IQR = 32.8–61.9 h). The TTP of the true-bacteremia group was significantly shorter than that of the contamination group (*P <* 0.0001). No significant difference was observed for the true-bacteremia (*P =* 0.95) and contamination groups (*P =* 0.62) when comparing the median TTP of the RapID CB Plus to that of MALDI-TOF MS (S1 Table). The details of the collected data are provided in S2 and S3 Tables.

**Table 3:** Comparison of time to positivity in blood cultures.

Group	Time to positivity (h)					*P*
	Min	25%	Median	75%	Max	
Diagnosis group						
True-bacteremia group, *n* = 77	14.6	23.0	26.8	38.2	135.4	< 0.0001
Contamination group, *n* = 88	14.2	32.8	43.3	61.9	188.2	
History of antibiotic administration						
Antibiotic use group, *n* = 88	14.2	24.0	29.8	43.5	188.2	0.001
No antibiotic group, *n* = 77	17.0	30.0	41.0	56.7	151.9	
Group of *Corynebacterium*						
Non-lipophilic group, *n* = 122	14.2	24.0	30.6	43.2	135.4	0.0001
Lipophilic group, *n* = 26	21.7	33.5	47.7	79.6	188.2	

The ROC curve was generated under the condition that cases with below-threshold TTP were diagnosed as true bacteremia. The generated ROC curves are shown in S1 Fig. For all *Corynebacterium*, the area under the curve (AUC) was 0.780 (95% confidence interval (CI) = 0.707–0.853; Table 4). The TTP threshold for the maximum value of the sum of sensitivity and specificity was 31.2 h. When cases with TTP ≤ 31.2 h were diagnosed as true bacteremia, the sensitivity, and specificity were 70.1% and 81.8%, respectively (Accuracy = 0.76). When cases with TTP ≤ 25.0 h were diagnosed as true bacteremia, the sensitivity and specificity were 44.2% and 95.5%, respectively. When cases with TTP ≤ 69.4 h were diagnosed as true bacteremia, the sensitivity and specificity were 96.1% and 20.5% respectively.

**Table 4:** Performance of a model for diagnosing bacteremia using time to positivity.

*Corynebacterium* Group	Threshold (h) ^a^	Sensitivity ^a^	Specificity ^a^	Accuracy ^a^
All, *n* = 165	31.2	70.1%	81.8%	0.76
(AUC: 0.780, 95% CI: 0.707–0.853)	25.0	44.2%	95.5%	0.72
	69.4	96.1%	20.5%	0.56
Non-lipophilic, *n* = 122	30.1	77.4%	80.0%	0.79
(AUC = 0.806, 95% CI: 0.727–0.885)	23.8	37.1%	95.0%	0.66
	56.6	95.2%	25.0%	0.61
Lipophilic, *n* = 26	38.2	57.1%	83.3%	0.69
(AUC = 0.714, 95% CI: 0.511–0.917)	31.7	35.7%	91.7%	0.62
	131.0	92.9%	25.0%	0.62

^a^ Sensitivity, specificity, and accuracy are shown when the time to positivity result (h) is below the threshold to determine true bacteremia. Accuracy was calculated as [(sensitivity) × (prevalence) + (specificity) × (1—prevalence)].

Abbreviations: AUC, area under the curve; CI, confidence interval.

The sensitivity, specificity, and accuracy at each threshold for the non-lipophilic and lipophilic groups are listed in Table 4. The accuracy of the non-lipophilic group (0.79) was comparable to that of all Corynebacterium (0.76). The accuracy of the lipophilic group (0.69) was lower than that of all Corynebacterium (0.76) and the non-lipophilic group (0.79).

### Evaluation of antibiotic administration and *Corynebacterium* group

The median TTP of the antibiotic use group (*n* = 88) was 29.8 h (IQR 24.0–43.5 h), whereas that of the no antibiotic group (*n* = 77) was 41.0 h (IQR 30.0–56.7 h; Table 3). The TTP of the antibiotic use group was significantly shorter than that of the no antibiotic group (*P* = 0.001).

The median TTP of the non-lipophilic group (*n* = 122) was 30.6 h (IQR 24.0–43.2 h; Table 3). The median TTP of the lipophilic group (*n* = 26) was 47.7 h (IQR 33.5–79.6 h). The TTP of the non-lipophilic group was significantly shorter than that of the lipophilic group (*P* = 0.0001).

### Association between time to positivity and each factor

We conducted multivariate logistic regression analysis for 148 cases, excluding 17 cases in which lipophilic-unknown *Corynebacterium* was isolated. The results of the multivariate logistic regression analysis are summarized in S4 Table. The threshold was set at 31.2 h, at which the maximum value was obtained for the sum of sensitivity and specificity. The true-bacteremia group was associated with a TTP ≤ 31.2 h (odds ratio, 10.0; 95% CI, 4.24–23.80; *P <* 0.0001). The lipophilic group was also associated with a TTP ≤ 31.2 h (odds ratio, 0.08; 95% CI, 0.02–0.28; *P <* 0.0001). The antibiotic-use group was not independently associated with TTP ≤ 31.2 h (odds ratio, 1.99; 95% CI, 0.80–4.95; *P* = 0.14).

### True-bacteremia case with only one set of positive blood cultures

For the true-bacteremia group, 18.2% (14/77) of cases had only one set of positive blood cultures. For the true-bacteremia group with one set of positive blood cultures (n = 14), 10 cases had additional positive blood cultures, and the remaining 4 cases had the same *Corynebacterium* in blood cultures as that isolated from other specimens (2 urine, 1 catheter, and 1 sputum specimens; Table 5). Of the 14 cases, 3 cases showed a TTP ≤ 25.0 h, and no case showed a TTP > 69.4 h.

**Table 5:** True-bacteremia case with only one set of positive blood cultures.

No.	Age	Sex	Patient status	Antibiotic	Species	TTP (h)	Culture-positive sample
1	74	Male	Inpatient	No	*C*. *amycolatum*	55.0	Catheter
2	78	Male	Inpatient	Yes	*C*. *striatum*	34.6	Sputum
3	51	Male	Outpatient	No	*C*. *jeikeium*	27.6	Additional blood
4	54	Male	Inpatient	Yes	*C*. *striatum*	27.1	Additional blood
5	80	Female	Outpatient	No	*C*. *striatum*	68.2	Additional blood
6	82	Male	Outpatient	No	*C*. *striatum*	30.0	Urine
7	53	Female	Inpatient	Yes	*C*. *striatum*	24.0	Additional blood
8	54	Female	Inpatient	Yes	*C*. *striatum*	42.0	Additional blood
9	66	Female	Inpatient	Yes	*C*. *striatum*	24.0	Additional blood
10	72	Male	Inpatient	Yes	*C*. *striatum*	24.0	Additional blood
11	67	Male	Inpatient	No	*C*. *urealyticum*	32.6	Urine
12	75	Female	Inpatient	Yes	*C*. *striatum*	27.0	Additional blood
13	71	Female	Outpatient	Yes	*C*. *striatum*	59.9	Additional blood
14	62	Male	Inpatient	Yes	*C*. *jeikeium*	46.2	Additional blood

Antibiotic, history of antibiotic administration; TTP, time to positive.

## Discussion

*Corynebacterium* is not only a typical contaminating organism isolated from blood cultures but also a pathogen that primarily causes opportunistic infections [2, 11–13]. In this study, we investigated 165 cases in which *Corynebacterium* was isolated from blood, and the rate of true bacteremia was 46.7%. Previous studies have reported that the rate of true bacteremia among cases of *Corynebacterium* spp. was 9–52% [5, 9, 14]. The difference in the rate of true bacteremia among these studies may be related to the different patients, coryneform strains, definitions of true bacteremia, and contamination rates of blood cultures. In the early period of the study, the use of RapID CB Plus may have led to the inclusion of some cases in which the *Corynebacterium* species were not accurately identified. However, no significant difference was observed in the positive time between the RapID CB Plus and MALDI-TOF MS duration (true-bacteremia group, *P* = 0.95; contamination group, *P* = 0.62), which should have little impact on the TTP.

Previous studies have demonstrated that the TTP of the true-bacteremia group is shorter than that of the contamination group, indicating that TTP may help distinguish between true bacteremia and contamination [6–7, 9, 15]. Ruiz-Giardín *et al*. evaluated the TTP of the BacT/Alert system and reported median TTP of 12.7 and 20.6 h for the true-bacteremia and contamination groups, respectively [7]. In our study on *Corynebacterium*, the median TTP of the true-bacteremia group (26.8 h) was significantly shorter than that of the contamination group (43.3 h). The TTP in our study was longer than that in the study by Ruiz-Giardín *et al*. This discrepancy may be attributed to the different species of bacteria investigated. Our study included only *Corynebacterium*, although the Ruiz-Giardín *et al*. study included various bacterial species (e.g. Enterobacteriaceae and *Staphylococcus aureus*). Consequently, the TTP evaluated in our study may have been longer. Therefore, when interpreting the TTP of cases in which *Corynebacterium* is isolated, we need to consider the long TTP observed with *Corynebacterium* spp.

Distinguishing between true bacteremia and contamination is important, particularly in one-set positive cases. In the true-bacteremia group, 18.2% (14/77) of cases had only one set of positive blood cultures. Using the threshold TTP (31.2 h) where the sum of sensitivity and specificity was the highest, the sensitivity and specificity for diagnosing true bacteremia were 70.1% and 81.8%. This threshold is not sufficiently sensitive and specific to be applied in clinical practice for the differentiation of true bacteremia from contamination. No case with TTP > 69.4 h (threshold indicating sensitivity > 95%) was observed in the true-bacteremia group with one set of positive blood cultures. Therefore, if the TTP exceeded 69.4 hours, the possibility of true bacteremia was considered low. Reporting a long TTP from the clinical laboratory to the physician when the TTP exceeds 69.4 hours may help the physician distinguish between true bacteremia and contamination.

Only three cases showed TTP ≤ 25.0 h (threshold indicating specificity > 95%) in the true-bacteremia group with one set of positive blood cultures. Thus, excluding contamination using this threshold (25.0 h) would be difficult for a single set of blood culture positives. To distinguish true bacteremia from contamination, clinical and bacteriological data (signs, symptoms, underlying disease, presence or absence of medical devices, and number of positive blood-culture sets) are also important [16]. For the true-bacteremia cases that had only one set of positive blood cultures, considering clinical and bacteriological data, additional cultures were performed on blood or other specimens. The same *Corynebacterium* spp. as in blood were isolated from these specimens. Our results support the importance of performing an additional culture for one set of positive blood cultures when true bacteremia is suspected based on clinical and bacteriological data.

Antibiotic administration in the week prior to the bacteremia event reportedly delays TTP [7]. Conversely, in our study, the TTP of the antibiotic use group (median, 29.8 h) was shorter than that of the no antibiotic group (median, 41.0 h). This discrepancy may be related to the fact that *Corynebacterium* frequently causes true bacteremia in immunocompromised patients, which are typically treated using antibiotics. In our study, the rate of antibiotic use was higher in the true-bacteremia group (71.4%) than in the contamination group (37.5%), and the true-bacteremia group had a shorter TTP than the contamination group. These results show that the antibiotic use group might have more cases of true bacteremia and a shorter TTP than the no antibiotic group. Furthermore, the logistic regression analysis revealed that antibiotic use was not independently associated with the below-threshold TTP.

Lipophilic *Corynebacterium* grows slower than non-lipophilic *Corynebacterium* in standard broth and agar media. The TTP of the lipophilic group (median, 47.7 h) was longer than that of the non-lipophilic group (median, 30.6 h). Furthermore, the logistic regression analysis revealed that the non-lipophilic group was independently associated with the below-threshold TTP. A moderate change was observed in accuracy when comparing diagnostic models at thresholds that detect all *Corynebacterium* (31.2 h) and the non-lipophilic group only (23.8 h). Moreover, the diagnostic model at the lipophilic group threshold (38.2 h) showed low AUC. The AUC and accuracy may have decreased because of the small sample size of the lipophilic group (*n* = 26).

This study has several limitations. First, the amount of blood drawn for culture was not monitored. Appropriate blood volume sampling is recommended as an inadequate blood volume results in decreased pathogen detection rates [17–19]. If an appropriate amount of blood was not sampled, these cases might have had a longer TTP than the true TTP. In our study, blood volume may have affected the results. Second, this study was conducted at a single center. The accuracy of blood sampling volume is dependent on the sampling location and situation. For example, in situations such as emergency rooms or those with poor venous access, there may not be enough time for sampling and a lower volume of blood may be collected. Therefore, the TTP and cutoffs of this study may not be applicable in different settings.

In conclusion, TTP > 69.4 h is likely to indicate contamination, and this interpretation may help reduce unnecessary antibiotic administration for contamination cases. Meanwhile, in cases with ≤ 69.4 h TTP, diagnosing true bacteremia by TTP is difficult, and a diagnosis based on clinical and bacteriological data is more important. Regarding the applicability of TTP and cutoffs shown in this study to other settings, further investigation is needed for other sampling locations and situations.

## Supporting information

S1 Fig Receiver operating characteristic (ROC) curves for conditions in which cases with time to positive (TTP) below the threshold are diagnosed as true bacteremia. The numbers shown in the graph are the threshold times (specificity and sensitivity) at which the sum of the sensitivity and specificity is the maximum. A; ROC curves generated from TTP for all cases (n = 165), B; ROC curves generated from TTP of the non-lipophilic group (n = 122), C; ROC curves generated from TTP of the lipophilic group (n = 26).
(PDF)

S1 Table Comparing the median TTP of the RapID CB Plus to that of MALDI-TOF MS. (PDF)

S2 Table Data of patients. (PDF)

S3 Table Data of microbiological testing. (PDF)

S4 Table Factors associated with a TTP ≤ 31.2 h. (PDF)
