# Supplementary material for: Time to positivity of Corynebacterium in blood culture: Characteristics and diagnostic performance
Source: PLoS One. 2022 Dec 13;17(12):e0278595. doi: 10.1371/journal.pone.0278595 (PMC9747040; doi:10.1371/journal.pone.0278595)
Supplement: S1 Fig — The numbers shown in the graph are the threshold times (specificity and sensitivity) at which the sum of the sensitivity and specificity is the maximum. A; ROC curves generated from TTP for all cases (n = 165), B; ROC curves generated from TTP of the non-lipophilic group (n = 122), C; ROC curves generated from TTP of the lipophilic group (n = 26). (PDF) [file pone.0278595.s001.pdf]

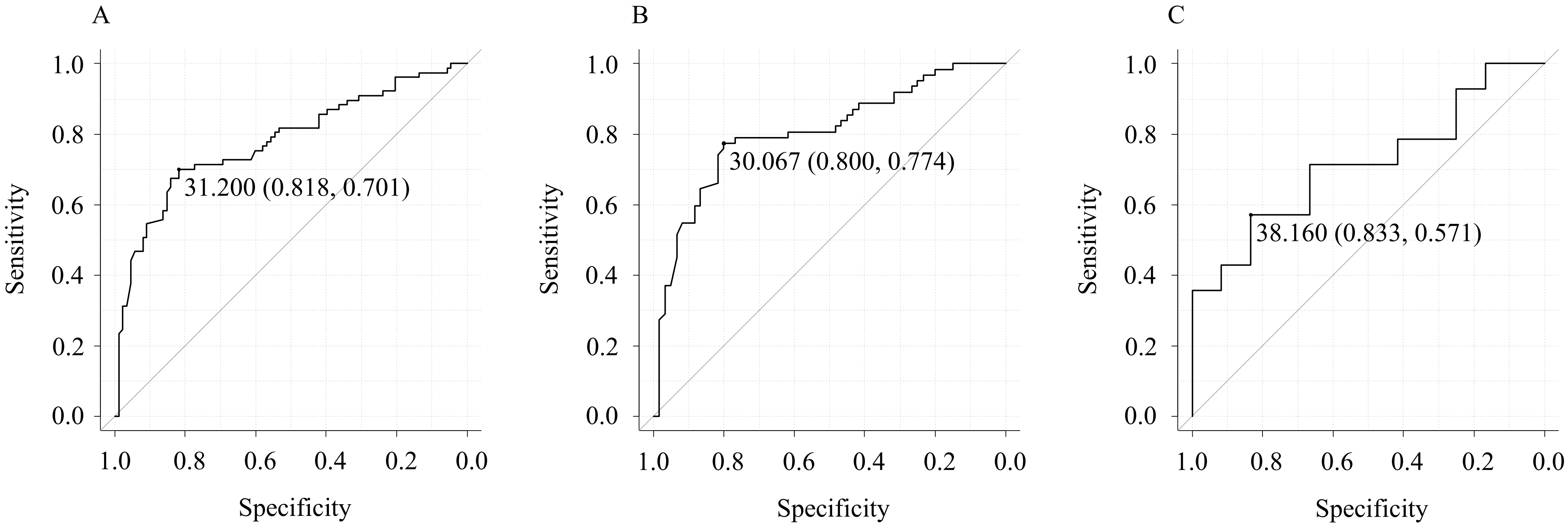

FIG S1 Receiver operating characteristic (ROC) curves for conditions in which cases with time to positive (TTP) below the threshold are diagnosed as true bacteremia. The numbers shown in the graph are the thresholds time (specificity and sensitivity) at which the sum of the sensitivity and specificity is the maximum. A; ROC curves generated from TTP for all cases (n=165), B; ROC curves generated from TTP of the non-lipophilic group (n=122), C; ROC curves generated from TTP of the lipophilic group (n=26).
