# Supplementary material for: Time to positivity of Corynebacterium in blood culture: Characteristics and diagnostic performance
Source: PLoS One. 2022 Dec 13;17(12):e0278595. doi: 10.1371/journal.pone.0278595 (PMC9747040; doi:10.1371/journal.pone.0278595)
Supplement: S1 Table — (PDF) [file pone.0278595.s002.pdf]

S1 Table. Comparing the median TTP of the RapID CB Plus to that of MALDI-TOF MS.

Group	Time to positive (h)					
	**Min**	**25th percentile**	**Median**	**75th percentile**	**Max**	***P***
True bacteremia group						
RapID CB Plus duration, n = 13	17.0	21.8	26.9	34.6	82.1	0.95
MALDI-TOF MS duration, n = 64	14.6	23.0	26.5	38.5	135.4	
Contamination group						
RapID CB Plus duration, n = 18	21.8	29.5	53.0	101.1	188.2	0.62
MALDI-TOF MS duration, n = 70	14.2	33.4	43.2	60.5	156.4	
