# Supplementary material for: Time to positivity of Corynebacterium in blood culture: Characteristics and diagnostic performance
Source: PLoS One. 2022 Dec 13;17(12):e0278595. doi: 10.1371/journal.pone.0278595 (PMC9747040; doi:10.1371/journal.pone.0278595)
Supplement: S2 Table — (PDF) [file pone.0278595.s003.pdf]

S2 Table. Data of patients

No.	Diagnosis group	Age	Sex	Inpatient or outpatient status	Department	History of antibiotic administration
1	Contamination	85	Male	Outpatient	Comprehensive internal medicine	No antibiotic
2	Contamination	84	Female	Outpatient	Emergency	No antibiotic
3	True bacteraemia	64	Female	Outpatient	Emergency	No antibiotic
4	True bacteraemia	36	Male	Inpatient	Hematology	Piperacillin-tazobactam, Cefepime
5	True bacteraemia	74	Male	Inpatient	Hematology	Piperacillin-tazobactam
6	Contamination	64	Male	Inpatient	Intensive care unit	Piperacillin-tazobactam
7	Contamination	45	Female	Outpatient	Gynecology	No antibiotic
8	Contamination	73	Male	Outpatient	Emergency	No antibiotic
9	Contamination	72	Male	Inpatient	Hematology	Piperacillin-tazobactam, Cefepime
10	Contamination	86	Male	Inpatient	Neurology	No antibiotic
11	Contamination	65	Female	Inpatient	Hematology	Piperacillin-tazobactam, Ampicillin
12	True bacteraemia	63	Male	Outpatient	Gastroenterology	No antibiotic
13	Contamination	72	Male	Outpatient	Emergency	No antibiotic
14	Contamination	80	Male	Inpatient	Respiratorology	No antibiotic
15	Contamination	70	Female	Inpatient	Hematology	No antibiotic
16	Contamination	60	Female	Outpatient	Emergency	Azithromycin
17	Contamination	67	Female	Inpatient	Respiratorology	Ampicillin-sulbactam
18	Contamination	71	Male	Inpatient	Spine surgery	No antibiotic
19	Contamination	76	Male	Outpatient	Emergency	No antibiotic
20	Contamination	41	Male	Inpatient	Cardiology	No antibiotic
21	True bacteraemia	74	Male	Inpatient	Nephrology	No antibiotic

S2 Table. Data of patients

No.	Diagnosis group	Age	Sex	Inpatient or outpatient status	Department	History of antibiotic administration
22	True bacteraemia	71	Female	Inpatient	Cardiology	Cefepime, Cefazolin
23	True bacteraemia	73	Male	Inpatient	Oncology	Piperacillin-tazobactam
24	Contamination	71	Male	Outpatient	Rheumatology and collagenology	No antibiotic
25	True bacteraemia	79	Female	Inpatient	Hematology	Trimethoprim-sulfamethoxazole
26	Contamination	4	Female	Outpatient	Pediatrics	No antibiotic
27	True bacteraemia	78	Male	Inpatient	Intensive care unit	Cefepime
28	True bacteraemia	90	Female	Outpatient	Emergency	No antibiotic
29	True bacteraemia	68	Male	Inpatient	Hematology	No antibiotic
30	True bacteraemia	51	Male	Outpatient	Nephrology	No antibiotic
31	True bacteraemia	54	Male	Inpatient	Hematology	Piperacillin-tazobactam
32	Contamination	68	Male	Inpatient	Comprehensive internal medicine	Cefazolin, Ampicillin-sulbactam
33	True bacteraemia	57	Female	Inpatient	Oncology	Cefepime
34	True bacteraemia	54	Male	Inpatient	Hematology	Piperacillin-tazobactam
35	True bacteraemia	74	Male	Inpatient	Hematology	Piperacillin-tazobactam
36	Contamination	42	Male	Inpatient	Neurology	Piperacillin-tazobactam
37	True bacteraemia	63	Male	Inpatient	Hematology	Meropenem
38	True bacteraemia	80	Female	Outpatient	Comprehensive internal medicine	No antibiotic
39	Contamination	78	Female	Outpatient	Emergency	No antibiotic
40	Contamination	69	Female	Outpatient	Emergency	No antibiotic
41	True bacteraemia	83	Female	Inpatient	Urology	No antibiotic
42	Contamination	60	Male	Outpatient	Emergency	No antibiotic

S2 Table. Data of patients

No.	Diagnosis group	Age	Sex	Inpatient or outpatient status	Department	History of antibiotic administration
43	Contamination	82	Male	Outpatient	Emergency	No antibiotic
44	Contamination	93	Male	Outpatient	Emergency	No antibiotic
45	Contamination	74	Female	Outpatient	Emergency	No antibiotic
46	True bacteraemia	84	Female	Outpatient	Comprehensive internal medicine	No antibiotic
47	True bacteraemia	62	Male	Outpatient	Emergency	Ciprofloxacin
48	True bacteraemia	72	Male	Inpatient	Hematology	Piperacillin-tazobactam
49	True bacteraemia	82	Male	Outpatient	Emergency	No antibiotic
50	Contamination	67	Male	Inpatient	Hematology	Trimethoprim-sulfamethoxazole
51	True bacteraemia	70	Female	Outpatient	Neurology	No antibiotic
52	True bacteraemia	72	Male	Inpatient	Hematology	Cefepime
53	Contamination	58	Male	Inpatient	Comprehensive internal medicine	No antibiotic
54	Contamination	96	Female	Inpatient	Plastic surgery	No antibiotic
55	Contamination	42	Male	Inpatient	Hematology	Levofloxacin
56	True bacteraemia	44	Male	Inpatient	Comprehensive internal medicine	No antibiotic
57	True bacteraemia	53	Female	Inpatient	Hematology	Piperacillin-tazobactam
58	True bacteraemia	77	Male	Inpatient	Hematology	Piperacillin-tazobactam
59	Contamination	90	Male	Inpatient	Comprehensive internal medicine	No antibiotic
60	True bacteraemia	78	Male	Inpatient	Hematology	No antibiotic
61	True bacteraemia	72	Male	Inpatient	Rheumatology and collagenology	Piperacillin-tazobactam, Ampicillin-sulbactam
62	True bacteraemia	73	Male	Inpatient	Hematology	Piperacillin-tazobactam
63	True bacteraemia	92	Male	Inpatient	Nephrology	No antibiotic

S2 Table. Data of patients

No.	Diagnosis group	Age	Sex	Inpatient or outpatient status	Department	History of antibiotic administration
64	True bacteraemia	95	Male	Inpatient	Gastroenterology	Piperacillin-tazobactam
65	Contamination	62	Male	Inpatient	Hematology	Piperacillin-tazobactam
66	Contamination	95	Male	Inpatient	Comprehensive internal medicine	Piperacillin-tazobactam
67	Contamination	77	Male	Outpatient	Emergency	No antibiotic
68	Contamination	65	Female	Inpatient	Hematology	Piperacillin-tazobactam
69	True bacteraemia	62	Male	Inpatient	Hematology	Piperacillin-tazobactam
70	Contamination	82	Female	Outpatient	Emergency	No antibiotic
71	True bacteraemia	20	Female	Inpatient	Hematology	Piperacillin-tazobactam
72	True bacteraemia	89	Female	Outpatient	Emergency	No antibiotic
73	Contamination	95	Male	Inpatient	Comprehensive internal medicine	Piperacillin-tazobactam
74	True bacteraemia	54	Female	Inpatient	Hematology	Meropenem
75	Contamination	74	Male	Inpatient	Digestive surgery	Cefazolin
76	Contamination	68	Male	Inpatient	Digestive surgery	Ampicillin-sulbactam
77	Contamination	66	Male	Outpatient	Emergency	No antibiotic
78	True bacteraemia	85	Female	Inpatient	Cardiology	No antibiotic
79	True bacteraemia	66	Female	Inpatient	Hematology	Trimethoprim-sulfamethoxazole
80	Contamination	61	Male	Outpatient	Emergency	No antibiotic
81	Contamination	89	Female	Outpatient	Emergency	No antibiotic
82	True bacteraemia	80	Male	Inpatient	Hematology	Piperacillin-tazobactam
83	Contamination	56	Female	Inpatient	Neurology	No antibiotic
84	True bacteraemia	92	Female	Outpatient	Emergency	Piperacillin-tazobactam

S2 Table. Data of patients

No.	Diagnosis group	Age	Sex	Inpatient or outpatient status	Department	History of antibiotic administration
85	Contamination	86	Male	Outpatient	Comprehensive internal medicine	No antibiotic
86	True bacteraemia	65	Male	Inpatient	Hematology	AZT
87	True bacteraemia	51	Female	Inpatient	Hematology	Meropenem, Piperacillin-tazobactam
88	True bacteraemia	62	Male	Inpatient	Hematology	Piperacillin-tazobactam
89	Contamination	72	Male	Inpatient	Hematology	Levofloxacin
90	Contamination	76	Male	Outpatient	Emergency	No antibiotic
91	True bacteraemia	67	Male	Inpatient	Hematology	Meropenem, Piperacillin-tazobactam
92	Contamination	78	Male	Outpatient	Emergency	No antibiotic
93	Contamination	56	Male	Inpatient	Neurology	No antibiotic
94	Contamination	84	Male	Outpatient	Emergency	No antibiotic
95	True bacteraemia	77	Male	Inpatient	Hematology	Levofloxacin
96	Contamination	71	Female	Inpatient	Hematology	No antibiotic
97	True bacteraemia	68	Male	Inpatient	Nephrology	No antibiotic
98	True bacteraemia	69	Male	Inpatient	Hematology	Piperacillin-tazobactam, Cefazolin
99	Contamination	89	Female	Outpatient	Emergency	No antibiotic
100	Contamination	81	Female	Outpatient	Emergency	No antibiotic
101	True bacteraemia	45	Male	Outpatient	Comprehensive internal medicine	No antibiotic
102	True bacteraemia	72	Male	Inpatient	Hematology	Piperacillin-tazobactam
103	True bacteraemia	66	Male	Inpatient	Hematology	Piperacillin-tazobactam
104	Contamination	71	Male	Outpatient	Emergency	No antibiotic
105	Contamination	25	Male	Outpatient	Emergency	No antibiotic

S2 Table. Data of patients

No.	Diagnosis group	Age	Sex	Inpatient or outpatient status	Department	History of antibiotic administration
106	Contamination	77	Female	Inpatient	Intensive care unit	No antibiotic
107	Contamination	69	Female	Inpatient	Respiratorology	No antibiotic
108	Contamination	74	Male	Inpatient	Oncology	No antibiotic
109	True bacteraemia	85	Male	Outpatient	Emergency	Meropenem
110	True bacteraemia	83	Male	Inpatient	Plastic surgery	Ampicillin-sulbactam
111	Contamination	81	Female	Outpatient	Emergency	No antibiotic
112	True bacteraemia	83	Female	Inpatient	Hematology	Piperacillin-tazobactam
113	True bacteraemia	86	Male	Inpatient	Digestive surgery	Ampicillin-sulbactam
114	Contamination	86	Male	Inpatient	Hematology	Piperacillin-tazobactam
115	Contamination	80	Male	Outpatient	Emergency	No antibiotic
116	True bacteraemia	67	Male	Inpatient	Urology	No antibiotic
117	True bacteraemia	82	Male	Inpatient	Urology	No antibiotic
118	True bacteraemia	77	Male	Inpatient	Respiratorology	Trimethoprim-sulfamethoxazole
119	True bacteraemia	65	Male	Inpatient	Intensive care unit	Piperacillin-tazobactam
120	True bacteraemia	74	Male	Inpatient	Oncology	No antibiotic
121	Contamination	86	Male	Outpatient	Emergency	No antibiotic
122	True bacteraemia	56	Male	Inpatient	Intensive care unit	Piperacillin-tazobactam
123	True bacteraemia	74	Male	Inpatient	Hematology	Piperacillin-tazobactam
124	Contamination	79	Male	Outpatient	Comprehensive internal medicine	No antibiotic
125	True bacteraemia	76	Male	Inpatient	Hematology	Piperacillin-tazobactam, Cefepime
126	Contamination	78	Female	Inpatient	Hematology	Cefepime

S2 Table. Data of patients

No.	Diagnosis group	Age	Sex	Inpatient or outpatient status	Department	History of antibiotic administration
127	Contamination	46	Male	Inpatient	Hematology	No antibiotic
128	Contamination	79	Male	Inpatient	Intensive care unit	Cefepime
129	True bacteraemia	68	Male	Inpatient	Hematology	Piperacillin-tazobactam, Cefepime
130	True bacteraemia	71	Male	Inpatient	Hematology	Piperacillin-tazobactam
131	Contamination	57	Male	Inpatient	Hematology	Piperacillin-tazobactam
132	Contamination	57	Male	Inpatient	Hematology	Meropenem
133	Contamination	57	Male	Inpatient	Hematology	Daptomycin
134	Contamination	70	Male	Outpatient	Emergency	No antibiotic
135	Contamination	64	Male	Inpatient	Hematology	No antibiotic
136	Contamination	91	Male	Inpatient	Comprehensive internal medicine	Cefepime
137	True bacteraemia	75	Female	Inpatient	Hematology	Piperacillin-tazobactam
138	Contamination	37	Male	Inpatient	Intensive care unit	Cefepime, Vancomycin
139	Contamination	40	Female	Inpatient	Intensive care unit	No antibiotic
140	Contamination	77	Female	Inpatient	Hematology	Meropenem, Piperacillin-tazobactam
141	True bacteraemia	72	Male	Inpatient	Hematology	Levofloxacin
142	Contamination	69	Male	Inpatient	Comprehensive internal medicine	Cefazolin
143	Contamination	67	Male	Inpatient	Intensive care unit	Meropenem, Trimethoprim-sulfamethoxazole
144	True bacteraemia	71	Female	Outpatient	Emergency	Piperacillin, Amoxicillin
145	Contamination	79	Male	Inpatient	Comprehensive internal medicine	No antibiotic
146	Contamination	64	Male	Inpatient	Intensive care unit	Vancomycin, Piperacillin-tazobactam
147	True bacteraemia	65	Male	Inpatient	Hematology	Cefepime

S2 Table. Data of patients

No.	Diagnosis group	Age	Sex	Inpatient or outpatient status	Department	History of antibiotic administration
148	True bacteraemia	77	Male	Inpatient	Hematology	Cefepime, Piperacillin-tazobactam
149	True bacteraemia	64	Male	Inpatient	Hematology	Meropenem, Metronidazole, Cefepime
150	Contamination	78	Male	Outpatient	Emergency	No antibiotic
151	Contamination	77	Female	Inpatient	Hematology	Levofloxacin
152	Contamination	46	Male	Inpatient	Hematology	Meropenem
153	True bacteraemia	62	Male	Inpatient	Hematology	Piperacillin-tazobactam
154	True bacteraemia	60	Male	Inpatient	Gastroenterology	Minocycline
155	True bacteraemia	68	Female	Inpatient	Hematology	Meropenem, Daptomycin
156	Contamination	85	Male	Outpatient	Emergency	No antibiotic
157	Contamination	42	Female	Outpatient	Emergency	No antibiotic
158	True bacteraemia	64	Female	Inpatient	Digestive surgery	No antibiotic
159	Contamination	63	Male	Inpatient	Cardiology	No antibiotic
160	True bacteraemia	90	Female	Inpatient	Comprehensive internal medicine	Piperacillin-tazobactam
161	Contamination	85	Male	Inpatient	Intensive care unit	Cefotiam
162	True bacteraemia	71	Male	Inpatient	Hematology	Piperacillin-tazobactam
163	Contamination	80	Male	Inpatient	Intensive care unit	Cefepime, Vancomycin, Ceftazidime
164	Contamination	98	Female	Outpatient	Intensive care unit	No antibiotic
165	Contamination	85	Male	Inpatient	Digestive surgery	Ampicillin-sulbactam
