# Supplementary material for: Time to positivity of Corynebacterium in blood culture: Characteristics and diagnostic performance
Source: PLoS One. 2022 Dec 13;17(12):e0278595. doi: 10.1371/journal.pone.0278595 (PMC9747040; doi:10.1371/journal.pone.0278595)
Supplement: S3 Table — (PDF) [file pone.0278595.s004.pdf]

S3 Table. Data of microbiological testing.

No.	Sets of blood cultures positive	Time to positeve (h)	Species	Group of Corynebacterium
1	1	50.9	*Corynebacterium striatum*	Non-lipophilic
2	1	38.4	*Corynebacterium afermentans*	Lipophilic-unknown
3	2	21.8	*Corynebacterium striatum*	Non-lipophilic
4	2	82.1	*Corynebacterium jeikeium*	Lipophilic
5	2	20.2	*Corynebacterium striatum*	Non-lipophilic
6	1	27.1	*Corynebacterium striatum*	Non-lipophilic
7	1	32.9	*Corynebacterium minutissimum*	Non-lipophilic
8	1	151.9	Other Coryneform bacteria	Lipophilic-unknown
9	1	27.1	*Corynebacterium striatum*	Non-lipophilic
10	1	115.0	*Brevibacterium casei*	Non-lipophilic
11	1	188.2	*Corynebacterium tuberculostearicum*	Lipophilic
12	2	26.9	*Corynebacterium striatum*	Non-lipophilic
13	1	110.4	*Corynebacterium striatum*	Non-lipophilic
14	1	55.2	*Brevibacterium* sp	Non-lipophilic
15	1	32.2	Other Coryneform bacteria	Lipophilic-unknown
16	1	24.0	*Corynebacterium striatum*	Non-lipophilic
17	1	60.7	*Corynebacterium jeikeium*	Lipophilic
18	1	62.2	*Corynebacterium minutissimum*	Non-lipophilic
19	1	28.6	Other Coryneform bacteria	Lipophilic-unknown
20	1	21.8	*Corynebacterium striatum*	Non-lipophilic
21	1	55.0	*Corynebacterium amycolatum*	Non-lipophilic
22	2	29.0	*Corynebacterium striatum*	Non-lipophilic

S3 Table. Data of microbiological testing.

No.	Sets of blood cultures positive	Time to positeve (h)	Species	Group of Corynebacterium
23	2	25.2	*Corynebacterium striatum*	Non-lipophilic
24	1	73.2	*Brevibacterium casei*	Non-lipophilic
25	2	20.6	*Corynebacterium striatum*	Non-lipophilic
26	1	119.0	Other Coryneform bacteria	Lipophilic-unknown
27	1	34.6	*Corynebacterium striatum*	Non-lipophilic
28	2	64.8	*Trueperella bernardiae*	Non-lipophilic
29	2	17.0	*Corynebacterium striatum*	Non-lipophilic
30	1	27.6	*Corynebacterium jeikeium*	Lipophilic
31	2	23.0	*Corynebacterium striatum*	Non-lipophilic
32	1	33.4	*Corynebacterium striatum*	Non-lipophilic
33	2	31.2	*Corynebacterium striatum*	Non-lipophilic
34	1	27.1	*Corynebacterium striatum*	Non-lipophilic
35	2	20.2	*Corynebacterium striatum*	Non-lipophilic
36	1	35.3	*Corynebacterium striatum*	Non-lipophilic
37	2	38.2	*Corynebacterium jeikeium*	Lipophilic
38	1	68.2	*Corynebacterium striatum*	Non-lipophilic
39	1	86.9	*Brevibacterium casei*	Non-lipophilic
40	1	33.6	*Dermabacter hominis*	Non-lipophilic
41	2	39.6	*Corynebacterium striatum*	Non-lipophilic
42	1	43.7	*Dermabacter hominis*	Non-lipophilic
43	1	41.0	*Corynebacterium amycolatum*	Non-lipophilic
44	1	33.4	*Corynebacterium aurimucosum*	Non-lipophilic

S3 Table. Data of microbiological testing.

No.	Sets of blood cultures positive	Time to positeve (h)	Species	Group of Corynebacterium
45	1	38.6	*Corynebacterium amycolatum*	Non-lipophilic
46	2	45.4	*Brevibacterium ravenspurgense*	Non-lipophilic
47	2	16.1	*Corynebacterium striatum*	Non-lipophilic
48	2	38.2	*Corynebacterium jeikeium*	Lipophilic
49	1	30.0	*Corynebacterium striatum*	Non-lipophilic
50	1	50.2	*Corynebacterium afermentans*	Lipophilic-unknown
51	2	26.9	*Corynebacterium riegelii*	Non-lipophilic
52	2	21.1	*Corynebacterium striatum*	Non-lipophilic
53	1	44.2	*Corynebacterium striatum*	Non-lipophilic
54	1	121.9	*Trueperella bernardiae*	Non-lipophilic
55	1	43.2	*Corynebacterium striatum*	Non-lipophilic
56	2	19.7	*Corynebacterium striatum*	Non-lipophilic
57	1	24.0	*Corynebacterium striatum*	Non-lipophilic
58	2	26.2	*Corynebacterium striatum*	Non-lipophilic
59	1	37.0	*Corynebacterium striatum*	Non-lipophilic
60	2	20.2	*Corynebacterium striatum*	Non-lipophilic
61	2	16.1	*Corynebacterium striatum*	Non-lipophilic
62	2	24.0	*Corynebacterium striatum*	Non-lipophilic
63	2	23.0	*Corynebacterium striatum*	Non-lipophilic
64	2	25.0	*Corynebacterium striatum*	Non-lipophilic
65	1	81.1	*Corynebacterium jeikeium*	Lipophilic
66	1	65.3	*Corynebacterium striatum*	Non-lipophilic

S3 Table. Data of microbiological testing.

No.	Sets of blood cultures positive	Time to positeve (h)	Species	Group of Corynebacterium
67	1	36.2	*Dermabacter hominis*	Non-lipophilic
68	1	27.1	Other Coryneform bacteria	Lipophilic-unknown
69	2	24.0	*Corynebacterium striatum*	Non-lipophilic
70	1	25.2	*Dermabacter hominis*	Non-lipophilic
71	2	15.1	*Corynebacterium striatum*	Non-lipophilic
72	2	21.6	*Corynebacterium striatum*	Non-lipophilic
73	1	81.8	*Corynebacterium simulans*	Non-lipophilic
74	1	42.0	*Corynebacterium striatum*	Non-lipophilic
75	1	31.7	*Corynebacterium jeikeium*	Lipophilic
76	1	14.2	*Corynebacterium striatum*	Non-lipophilic
77	1	41.8	*Corynebacterium singulare*	Non-lipophilic
78	2	69.4	Other Coryneform bacteria	Lipophilic-unknown
79	1	24.0	*Corynebacterium striatum*	Non-lipophilic
80	1	58.3	Other Coryneform bacteria	Lipophilic-unknown
81	1	55.7	*Corynebacterium afermentans*	Lipophilic-unknown
82	2	16.1	*Corynebacterium striatum*	Non-lipophilic
83	1	27.1	*Corynebacterium striatum*	Non-lipophilic
84	2	40.6	*Corynebacterium striatum*	Non-lipophilic
85	1	32.6	*Dermabacter hominis*	Non-lipophilic
86	2	135.4	*Corynebacterium jeikeium*	Lipophilic
87	2	23.0	*Corynebacterium striatum*	Non-lipophilic
88	2	49.2	*Corynebacterium jeikeium*	Lipophilic

S3 Table. Data of microbiological testing.

No.	Sets of blood cultures positive	Time to positeve (h)	Species	Group of Corynebacterium
89	1	32.2	*Corynebacterium striatum*	Non-lipophilic
90	1	37.7	*Corynebacterium amycolatum*	Non-lipophilic
91	2	29.5	*Corynebacterium striatum*	Non-lipophilic
92	1	30.0	*Corynebacterium simulans*	Non-lipophilic
93	1	78.0	*Corynebacterium afermentans*	Lipophilic-unknown
94	1	96.0	Other Coryneform bacteria	Lipophilic-unknown
95	2	31.2	*Corynebacterium jeikeium*	Lipophilic
96	1	79.2	*Corynebacterium mucifaciens*	Non-lipophilic
97	2	29.5	*Corynebacterium striatum*	Non-lipophilic
98	2	25.2	*Corynebacterium striatum*	Non-lipophilic
99	1	45.1	Other Coryneform bacteria	Lipophilic-unknown
100	1	25.7	*Corynebacterium aurimucosum*	Non-lipophilic
101	2	56.6	*Arcanobacterium haemolyticum*	Non-lipophilic
102	1	24.0	*Corynebacterium striatum*	Non-lipophilic
103	2	26.2	*Corynebacterium striatum*	Non-lipophilic
104	1	38.2	*Corynebacterium striatum*	Non-lipophilic
105	1	36.2	*Corynebacterium coyleae*	Non-lipophilic
106	1	23.8	*Corynebacterium striatum*	Non-lipophilic
107	1	56.7	*Corynebacterium afermentans*	Lipophilic-unknown
108	1	30.7	*Corynebacterium minutissimum*	Non-lipophilic
109	2	42.6	*Corynebacterium striatum*	Non-lipophilic
110	2	30.1	*Corynebacterium striatum*	Non-lipophilic

S3 Table. Data of microbiological testing.

No.	Sets of blood cultures positive	Time to positeve (h)	Species	Group of Corynebacterium
111	1	32.2	*Rothia dentocariosa*	Non-lipophilic
112	2	14.6	*Corynebacterium striatum*	Non-lipophilic
113	2	17.7	*Corynebacterium striatum*	Non-lipophilic
114	1	43.2	*Corynebacterium striatum*	Non-lipophilic
115	1	43.4	*Corynebacterium singulare*	Non-lipophilic
116	1	32.6	*Corynebacterium urealyticum*	Lipophilic
117	2	46.6	*Corynebacterium striatum*	Non-lipophilic
118	2	21.7	*Corynebacterium jeikeium*	Lipophilic
119	2	26.8	*Corynebacterium striatum*	Non-lipophilic
120	2	131.0	*Corynebacterium jeikeium*	Lipophilic
121	1	49.2	*Corynebacterium striatum*	Non-lipophilic
122	2	23.3	*Corynebacterium striatum*	Non-lipophilic
123	2	68.1	*Corynebacterium jeikeium*	Lipophilic
124	1	48.6	*Corynebacterium striatum*	Non-lipophilic
125	2	20.1	*Corynebacterium striatum*	Non-lipophilic
126	1	36.1	*Corynebacterium resistens*	Lipophilic
127	1	50.1	*Corynebacterium tuberculostearicum*	Lipophilic
128	1	40.1	*Corynebacterium striatum*	Non-lipophilic
129	2	18.2	*Corynebacterium striatum*	Non-lipophilic
130	2	41.7	*Corynebacterium striatum*	Non-lipophilic
131	1	132.2	*Corynebacterium tuberculostearicum*	Lipophilic
132	1	66.2	*Corynebacterium striatum*	Non-lipophilic

S3 Table. Data of microbiological testing.

No.	Sets of blood cultures positive	Time to positeve (h)	Species	Group of Corynebacterium
133	1	34.1	*Corynebacterium striatum*	Non-lipophilic
134	1	42.1	*Corynebacterium amycolatum*	Non-lipophilic
135	1	44.1	*Corynebacterium jeikeium*	Lipophilic
136	1	96.3	*Corynebacterium striatum*	Non-lipophilic
137	1	27.0	*Corynebacterium striatum*	Non-lipophilic
138	1	60.6	*Corynebacterium afermentans*	Lipophilic-unknown
139	1	61.9	Other Coryneform bacteria	Lipophilic-unknown
140	1	75.2	*Corynebacterium resistens*	Lipophilic
141	2	23.1	*Corynebacterium striatum*	Non-lipophilic
142	1	61.4	*Corynebacterium striatum*	Non-lipophilic
143	1	60.2	*Corynebacterium resistens*	Lipophilic
144	1	59.9	*Corynebacterium striatum*	Non-lipophilic
145	1	135.4	*Corynebacterium striatum*	Non-lipophilic
146	1	156.4	*Corynebacterium resistens*	Lipophilic
147	2	24.1	*Corynebacterium striatum*	Non-lipophilic
148	2	24.1	*Corynebacterium striatum*	Non-lipophilic
149	2	30.1	*Corynebacterium resistens*	Lipophilic
150	1	43.8	Other Coryneform bacteria	Lipophilic-unknown
151	1	58.1	*Corynebacterium amycolatum*	Non-lipophilic
152	1	35.1	*Corynebacterium striatum*	Non-lipophilic
153	1	46.2	*Corynebacterium jeikeium*	Lipophilic
154	2	24.6	*Corynebacterium jeikeium*	Lipophilic

S3 Table. Data of microbiological testing.

No.	Sets of blood cultures positive	Time to positeve (h)	Species	Group of Corynebacterium
155	2	28.2	*Corynebacterium striatum*	Non-lipophilic
156	1	66.8	*Dermabacter hominis*	Non-lipophilic
157	1	32.8	*Dermabacter hominis*	Non-lipophilic
158	2	28.7	*Corynebacterium striatum*	Non-lipophilic
159	1	26.8	*Corynebacterium minutissimum*	Non-lipophilic
160	2	15.7	*Corynebacterium striatum*	Non-lipophilic
161	1	25.8	*Corynebacterium striatum*	Non-lipophilic
162	2	24.2	*Corynebacterium striatum*	Non-lipophilic
163	1	42.8	*Corynebacterium jeikeium*	Lipophilic
164	1	30.6	*Corynebacterium amycolatum*	Non-lipophilic
165	1	44.3	*Corynebacterium striatum*	Non-lipophilic
