# Supplementary material for: Time to positivity of Corynebacterium in blood culture: Characteristics and diagnostic performance
Source: PLoS One. 2022 Dec 13;17(12):e0278595. doi: 10.1371/journal.pone.0278595 (PMC9747040; doi:10.1371/journal.pone.0278595)
Supplement: S4 Table — (PDF) [file pone.0278595.s005.pdf]

S4 Table. Factors associated with a TTP ≤ 31.2 h.

Factor	Odds ratio (95% CI)	*P*
**Step1**		
Age	0.99 (0.96-1.01)	0.3
Sex	1.00 (0.38-2.59)	0.99
Department	1.06 (0.94-1.20)	0.32
Diagnosis group, true bacteremia group	10.00 (4.20-24.00)	< 0.0001
Corynebacterium group, lipophilic group	0.08 (0.02-0.28)	< 0.001
History of antibiotic administration, antibiotic use group	1.99 (0.79-5.03)	0.14
**Step2**		
Age	0.99 (0.96-1.01)	0.3
Department	1.06 (0.94-1.20)	0.32
Diagnosis group, true bacteremia group	10.00 (4.24-23.80)	< 0.0001
Corynebacterium group, lipophilic group	0.08 (0.02-0.28)	< 0.0001
History of antibiotic administration, antibiotic use group	1.99 (0.80-4.95)	0.14

The P value of sex was >0.5 in step 1. Therefore, step 2 was performed excluding sex.
